# Characterizing Sleep Issues Using Twitter

**DOI:** 10.2196/jmir.4476

**Published:** 2015-06-08

**Authors:** David J McIver, Jared B Hawkins, Rumi Chunara, Arnaub K Chatterjee, Aman Bhandari, Timothy P Fitzgerald, Sachin H Jain, John S Brownstein

**Affiliations:** ^1^ Boston Children's Hospital, Harvard Medical School Boston, MA United States; ^2^ New York University New York, NY United States; ^3^ Merck & Co, Inc Boston, MA United States; ^4^ Merck & Co, Inc West Point, PA United States; ^5^ CareMore Health System Cerritos, CA United States

**Keywords:** sleep issues, social media, insomnia, novel methods, sentiment, depression

## Abstract

**Background:**

Sleep issues such as insomnia affect over 50 million Americans and can lead to serious health problems, including depression and obesity, and can increase risk of injury. Social media platforms such as Twitter offer exciting potential for their use in studying and identifying both diseases and social phenomenon.

**Objective:**

Our aim was to determine whether social media can be used as a method to conduct research focusing on sleep issues.

**Methods:**

Twitter posts were collected and curated to determine whether a user exhibited signs of sleep issues based on the presence of several keywords in tweets such as insomnia, “can’t sleep”, Ambien, and others. Users whose tweets contain any of the keywords were designated as having self-identified sleep issues (sleep group). Users who did not have self-identified sleep issues (non-sleep group) were selected from tweets that did not contain pre-defined words or phrases used as a proxy for sleep issues.

**Results:**

User data such as number of tweets, friends, followers, and location were collected, as well as the time and date of tweets. Additionally, the sentiment of each tweet and average sentiment of each user were determined to investigate differences between non-sleep and sleep groups. It was found that sleep group users were significantly less active on Twitter (*P*=.04), had fewer friends (*P*<.001), and fewer followers (*P*<.001) compared to others, after adjusting for the length of time each user's account has been active. Sleep group users were more active during typical sleeping hours than others, which may suggest they were having difficulty sleeping. Sleep group users also had significantly lower sentiment in their tweets (*P*<.001), indicating a possible relationship between sleep and pyschosocial issues.

**Conclusions:**

We have demonstrated a novel method for studying sleep issues that allows for fast, cost-effective, and customizable data to be gathered.

## Introduction

In 2006, between 50-70 million adults in the United States had perceived chronic sleep or wakefulness issues, which is an increasing trend, and more than 35% of adults report having insufficient sleep [1]. With the most common sleep issues reported by Americans as having <7 hours of sleep in a 24-hour period, restless leg syndrome, snoring, and insomnia, there are many areas where further exploration could be beneficial [1]. These forms of sleep-depriving conditions have been linked to decreased quality of life, excessive daytime sleepiness, depression, obesity, cardiovascular complications, diabetes, decreased productivity, increased chance of risky behaviors, increased risk of car accidents, and others [2-5]. Impaired sleeping can lead to serious impact on health; for example, the US Department of Transportation found that 2.2-2.6% of all fatal car crashes from 2005-2009 reportedly involved drowsy driving [6]. As well, depression has been an area of active research in attempting to determine its role in insomnia and sleep disorders, in either causal direction [7-11]. Due to the impact, both physical and psychosocial, of sleep-related issues on a large segment of the population, continued research in this area is needed.

For decades, interest in sleep issues has produced broad research and survey methods. In addition to studies and surveys being undertaken by private organizations such as the National Sleep Foundation, the Centers for Disease Control and Prevention (CDC), via the Behavioral Risk Factor Surveillance System (BRFSS), administers yearly questionnaires to the American, non-incarcerated population, regarding many types of health and risk factors. Beginning in 2009, the BRFSS has included a module dealing exclusively with sleep issues [1]. While the data gathered by the BRFSS have been instrumental in our understanding of sleep disorders, it does suffer from several limitations. The BRFSS is based on a random-digit-dialing system, and response rates can be low. Of all calls made, a response rate of between 40-67%, while respectable for epidemiological surveys, means much of the intended population is not being surveyed [1], though sample size and weighting calculations can correct for some of this bias. Of note, not all US states are included in the survey each year; therefore, the generalizability of the results to the entire US population is negatively impacted. Finally, because of the monumental amount of work involved in performing the surveys, gathering and combining data, analysis, and publication, the resulting BRFSS reports are expensive and are typically 7 months old by the time they are released. There have been many investigations of sleep disorders by independent researchers, but they too tend to suffer from some of these limitations, such as small sample size [12], high cost [13], long time frames [14], and lack of generalizability [15]. In light of these shortcomings, new supplemental methods of investigating the epidemiologic factors associated with sleep issues are needed to provide timely analyses that have greater external validity by incorporating a much larger sample size, and which are less costly, more quickly implemented and analyzed, and are malleable to allow for design restructuring based on new data.

We are interested in determining whether the way in which people with potential sleep issues interact with Twitter can be used as a method of identifying and characterizing those individuals. In recent years, there has been a great deal of interest in harnessing the massive amounts of data produced by social media websites, such as Facebook and Twitter, to try to glean insights into topics of interest to public health, and these platforms are increasingly being considered as valuable sources of patient information [16-19]. Recent examples include using social media to perform infectious and foodborne disease surveillance [20-22], chronic disease surveillance [23], prescription drug use [24], investigating hospital care quality [25], and many others [26,27]. With a greater focus on human behavior and characterization, researchers have used Twitter to investigate how people use social media in efforts of weight loss [28] and how suicide-related Twitter use compares to actual events [29]. Additionally, an increasing number of researchers have been experimenting with sentiment analysis on social media [30-35]. Sentiment can be determined in several ways, with the principle being to classify the underlying emotional information (within tweets, status updates, photos, etc) as either positive or negative; this can be done either purely by human input or by an algorithm trained to complete this process based on a human-classified set of objects. This process is useful for determining how people feel about products, events, other people, etc. Sentiment analysis has yet to be used on social media to help understand sleep disorders, but it does exhibit a diurnal characteristic [30] and offers interesting possibilities in investigating the links between sleep disorders and the overall sentiment or attitude of individuals displaying these characteristics. Demographics of Twitter users, while not entirely representative of the American population, have become more representative over time. Twitter is currently used by 23% of the adult Internet-using population and has seen increases in usage from hitherto underrepresented populations, such as men, whites, people aged 65 and older, and others. As of late 2014, 24% and 21%, respectively, of male and female adult Internet users used Twitter, and only 37% of that group were under 30 years of age [36].

We were interested in finding out if people who posted on Twitter about having sleep issues were more active on Twitter than people who did not, or if they had more friends or followers. As well, we wanted to know if people discussing sleep issues were posting more during traditional sleeping hours, suggesting that they may be having difficulty with sleeping. Furthermore, we were interested in the relationship between users who exhibited potential sleep issues and the sentiment of what it was they were tweeting, as a means of exploring the impact of sleep issues on emotions, feelings, and attitudes.

In this study, information posted on Twitter was used to identify people who may be exhibiting self-described signs or symptoms of sleep-related issues. By examining the content of tweets, users whose tweets contained specific sleep-related keywords were compared to a random population that did not contain these keywords. We then examined if there were observable differences between these groups in relation to their activity on Twitter.

## Methods

### Overview

Twitter is an online microblogging website where users “tweet”, or post, statuses containing 140 characters or less. It boasts approximately 255 million monthly active users worldwide, including 33% residing in the United States [37]. Twitter allows conditional access to this wealth of information through their application programming interface (API), for data that users allow to be public. Using the Twitter API, one can collect tweets matching certain query criteria and access meta information including location (self-reported and geo-tagged), total number of tweets, number of “followers”, number of friends, etc.

Twitter users who mentioned pre-defined keywords related to sleep or sleep issues in their tweets (sleep group) were compared to users whose tweets did not contain pre-defined keywords (non-sleep group). Sleep group tweets were identified on the basis of keywords being present in a curated tweet, on a prospective basis, starting on January 7, 2014, and ending on April 30, 2014, and were examined and curated on a “most recent tweet” basis. That is, during each curation session, tweets that were most recently posted to Twitter were analyzed first.

To build a corpus of both sleep group and non-sleep group users, code was written to access the Twitter API, which searched Twitter every 15 minutes for all new tweets containing any of the following keywords: “can’t sleep”, “insomnia”, “melatonin”, “Ambien”, “Ambien-CR”, “zolpidem”, “Lunesta”, “Intermezzo”, “trazadone”, “eszopiclone”, “#teamnosleep”, and “#cantsleep” (note that “#” is the symbol for a Twitter hashtag that denotes a user-identified topic within the tweet, and “teamnosleep” is a user-created hash tag often used by individuals who declare that they are unable to sleep). The list of Twitter search terms was identified through consultation with researchers with expertise in sleep-related fields of study and by experimentally querying the Twitter database to investigate which terms were most commonly used. By including keywords and hashtags that are related to specific medications (ie, zolpidem, Intermezzo, eszopiclone), we aimed to collect tweets that we were highly confident would be related to some type of sleep issue, even if the number collected was small. In contrast, by including keywords and hashtags that were broader (sleep, tired, insomnia, etc), we hoped to collect a large number of tweets, but not all of which would be strictly relevant. Since all tweets included in the study were manually curated, the low specificity of tweets collected under the more generic keywords was not an issue. This was not an exhaustive search across all possible search terms, but rather an exploratory approach to test the utility of this type of analysis.

To assess authenticity and ensure they met sleep group inclusion criteria, tweets that contained one or more of these keywords were manually curated, by a single individual (DM), looking for the following attributes. To be included as sleep group tweets, a tweet (and the Twitter account it is associated with) (1) must have been in the English language (as selected in user settings), (2) appeared to be from within the United States, (3) be owned by an “average” person (ie, not a company/corporation, celebrity, or spam account), and (4) was not a “re-tweet” (a re-post of a tweet originally posted by a different user). Re-tweets were removed because we were interested only in the experiences and expressed feelings of the individuals we were collecting information on, and not those of other people. Twitter accounts were qualitatively determined to be within the United States if the user-defined location was set to a US location or the account appeared to be located in the United States based on the nature of the user’s profile information and previous posts. As well, tweets were examined to ensure that the keyword selected in the tweet was being used in the proper context. For example, a tweet that read “Just took my Ambien, hope I can sleep tonight” would be accepted as a sleep group user, but the tweet “A friend of mine just got prescribed Ambien” would not, because it did not pertain to the person who issued the tweet. Similarly, tweets that were ambiguous as to whether or not an action or outcome pertained to the individual who wrote the tweet were not treated as sleep group users. For example, the tweet “I took an Ambien, and now I’m sleepy” would be treated as a sleep group user, but the tweet “Ambien makes you sleepy” would not, because it did not indicate that this person took Ambien or was sleepy. They were simply making a statement.

A corpus of potential non-sleep group tweets was built by collecting tweets that did not contain any of the pre-defined keywords of interest. After initial manual curation to ensure tweets and users were in the English language, were from the United States, and were “normal” users, users were added to the non-sleep group if none of their tweets within the previous 10 days contained any of the pre-defined keywords of interest; text found in re-tweets was not considered. As an introductory and exploratory study, 10 days was chosen as a number of days that would allow for enough tweets to provide sufficient data for our purposes and was both computationally and financially achievable.

Tweets were automatically collected on an ongoing basis and selection of users into either the sleep group or non-sleep group was performed by the curator on a “most recent tweet” basis. That is, when the curator logged on to the curating tool, the most recent tweets to be collected were presented for curation. Therefore, if the curator were curating tweets at 9 am EST, the tweets they would be working on were the most recent tweets posted that matched the search criteria.

### User Data

User-related data are data that are associated with a user’s Twitter account as opposed to a particular tweet. For each user curated and included in the study, the metadata included in the analyses were total number of tweets, number of favorites (number of times that user favorited tweets from other users), total number of followers, total number of friends, user-submitted location, date of account creation, time zone of user, average number of tweets per day since account creation (calculated as total number of tweets divided by number of days that account has been active). For several of these collected variables, the count of the variable was also averaged over the lifetime of the user’s account. This was done by dividing the variable count by the number of days the user had been active, which is equal to the number of days between account creation and the day the identified tweet was written. By creating data for the average number of counts/actions per day, the fact that some users have a higher number of friends, followers, or status updates, simply because they have had a Twitter account longer than some other users was accounted for. We also calculated the ratio of Twitter followers to friends for each user to create a way of measuring influence or impact on Twitter; a high follower:friend ratio indicates that a user has many people who follow their account but that they themselves follow relatively few people. This is often an indicator of high-impact Twitter users [38] and was included to ensure that both sleep- and non-sleep groups were equal in this respect.

To ensure that user data were collected at the same time for all study users, user metadata was collected after all tweets had been identified, rather than at the time of tweet approval. This was done primarily due to the increased time it took to identify sleep group tweets as compared to non-sleep group tweets. As a result, user metadata and tweet data presented in this study represent the state of a user’s account as of May 1, 2014.

### Tweet/Timeline Data

Tweet data are the data associated with a single tweet as opposed to the data associated with the user who issued the tweet. For each tweet that was included in the study, the analyzed tweet metadata included 140-character (maximum) tweet text, date and time of tweet creation (in Universal Time Code, UTC), and geo-tagged location of tweet (when available).

Similar to parsing non-sleep group users’ previous 10 days of activity to search for keywords, additional information was gathered on all users to investigate the overall trend of non-sleep group users’ behavior versus sleep group users’ tweeting behavior. From the original tweet that was manually curated to classify a user, a minimum of 10 days’ worth of previous tweets were collected from a user’s timeline. The process proceeded such that the Twitter API was queried to return 200 tweets for a given user. If the returned 200 tweets represented less than 10 days’ worth of tweets, the process was repeated until 10 days of tweets were collected, or until the Twitter API indicated that the user had no more data to retrieve.

For all study users, the number of tweets that were published during certain times of day (coded as 1: midnight-5:59 am, 2: 6 am-11:59 am, 3: 12 pm-5:59 pm, 4: 6 pm-11:59 am) and on which day of the week they were created was determined. All tweet times used in this analysis were converted from UTC to the user’s local time (based on the user’s time zone). While it is possible that a user has an incorrect time zone set, this is highly unlikely as it is based on the time zone of their computer or smart device.

### Sentiment Analysis

To determine the difference in sentiment of tweets published by sleep group users and non-sleep group users, Amazon’s Mechanical Turk (AMT) platform was used. Amazon’s Mechanical Turk is an online tool that allows large, tedious jobs to be completed very quickly by harnessing the efforts of numerous personnel hired by Amazon [39]. For this study, we had AMT workers perform a sentiment analysis on select tweets. This is a popular AMT feature in which text (in this case tweets) is rated as having either a strongly positive, positive, neutral, negative, or strongly negative sentiment (recorded as 2, 1, 0, -1, -2, respectively). Ratings are of course based on each AMT worker’s own subjective opinion. For each Twitter user included in the study, 20 of their tweets (the original, curated tweet plus the user’s previous 19 tweets) were rated by AMT workers, in a randomized, de-identified, non-categorized format. Two AMT employees, who were classified by Amazon as being highly experienced in the field of sentiment analysis (Master Workers) [40], rated each tweet. The result was an average sentiment score for each tweet, across both sleep and non-sleep groups. Because only two users rated each tweet, the final average sentiment results were grouped into the following categories: Positive=0.5, 1.0, 1.5, 2.0; Neutral=0; Negative=-0.5, -1.0, -1.5, -2.0. By comparing the proportion of sleep group and non-sleep group tweets that were identified as positive, negative, or neutral, sentiment differences were assessed. While there are numerous software options for determining the sentiment of any string of text (such as tweets), we opted to use AMT as it involves human graders, which is the gold standard on which many automated methods are based [41,42]. Humans are better able to catch uses of language, such as irony or sarcasm, that are difficult for computers to identify. In addition, while machines may be better at identifying individual words attributed to positive and negative sentences, determining the sentiment of a complex sentence and taking word context into consideration is still quite difficult for a machine [43].

To ensure that AMT workers were rating tweet sentiment reliably, we calculated agreement and Cohen’s kappa values between sets of workers. Because AMT can use hundreds of individual workers for a project, we focused our efforts on the AMT workers who were most prolific in rating tweet sentiment to capture at least 20% of rating jobs.

### Statistical Methods

To investigate differences between sleep group users and non-sleep group users for variables with highly skewed distributions, permutation analyses with 10,000 iterations with re-sampling was used to investigate differences in median values. Variables based on proportions, such as the proportion of a user’s tweets published on a certain day of the week, were compared between groups by performing two-tailed, two-group proportion tests, with statistical significance considered to be a *P* value of ≤.05. All analyses were performed in Stata 13.

### Code and Database Structure

Custom code was written in PHP (hypertext preprocessor) to access the Twitter REST API (v1.1), which utilizes the open-source OAuth library tmhOAuth. Tweets are accessed via the Twitter API as “status objects”, which are structured, JSON-formatted objects that contain all of the metadata about both the individual tweet and the user. Tweets were searched on the presence or absence of keywords using the GET search/tweets request. User timelines were collected using the GET statuses/user_timeline request. Returned tweets were stored in an Amazon Web Service (AWS) Relational Database Service (RDS) MySQL database as complete status objects in JSON format. Additionally, some tweet and user fields were stored in separate MySQL tables for faster access. Subsequent analysis and data cleaning were done using custom scripts written in PHP and Python.

## Results

As of May 1, 2014, the total number of sleep group tweets that were collected over 115 days and stored in the database was 2,820,427. The number of tweets collected for each keyword are reported in Table 1. Due to the large number of tweets collected, only a small percentage could be analyzed. Of all collected tweets, 1000 of both sleep group and non-sleep group users (N=2000) were manually curated and approved for inclusion in the study. At the time of user account metadata collection on May 1, 2014, there were some accounts that had become inaccessible (eg, switched to a private setting, deleted, or banned from Twitter). After accounting for these changes, our final dataset included 896 sleep group users and 934 non-sleep group users. Summary statistics of the collected user metadata and tweet data, categorized by user group, are presented in Table 2 and Table 3, respectively.

**Table 1:** Number of tweets collected by various insomnia or sleep related keywords.^a^

Keyword	n	Proportion, %
#TeamNoSleep	119,378	4.23
Ambien	54,420	1.93
Can't Sleep	1,533,704	54.38
Eszopiclone	151	0.01
Insomnia	994,049	35.24
Intermezzo	10,145	0.36
Lunesta	3,734	0.13
Melatonin	103,674	3.68
Trazadone	1,149	0.04
Zaleplon	23	0.00
Total	2,820,427	100.00

^a^Number of tweets collected per keyword in this list represent different forms and combinations of each keyword (ie, Can’t Sleep includes “Can’t Sleep” as well as “#cantsleep”) as well as re-tweeted tweets. Some tweets may contain more than one keyword.

**Table 2:** Twitter user data.

Variable		Total		Per day^a^	
		Mean	Median	Mean	Median
**Days active, n**					
	Non-sleep group	817	777		
	Sleep group	1054	993		
	*P* value		<.001		
**Favorites, n**					
	Non-sleep group	1909	684	4.8	1.1
	Sleep group	3257	1069	6.2	1.3
	*P* value		<.001		.11
**Followers, n**					
	Non-sleep group	817	319	5.5	0.5
	Sleep group	792	295	1.2	0.3
	*P* value		.08		<.001
**Friends, n**					
	Non-sleep group	689	318	6.4	0.5
	Sleep group	518	295	1.3	0.3
	*P* value		.13		<.001
**Follower:Friend ratio**					
	Non-sleep group	1.44	1.01		
	Sleep group	1.45	0.99		
	*P* value		0.901		
**Statuses, n**					
	Non-sleep group	12609	5853	22	10
	Sleep group	15253	7622	18	8
	*P* value		<.001		.04

^a^Per day data refers to the total count of the variable divided by the total number of days a user’s account has been active.

**Table 3:** Proportion of tweets posted at time of day by group.

	Proportion of tweets (%) by time			
	0:00-5:59	6:00-11:59	12:00-17:59	18:00-23:59
Non-sleep group	12.1	22.5	28.7	36.7
Sleep group	16.8	16.3	28.6	38.1
*P* value	<.001	<.001	.72	<.001

Sleep group users had Twitter accounts that were significantly older than other users (*P*<.001). The number of tweets overall were higher for users in the sleep group than for non-sleep group users (*P*<.001), but when calculated as the number of tweets per day since account creation, sleep group users had significantly fewer tweets (*P*=.04). The total number of tweets a user has favorited (other user’s tweets) was significantly higher for sleep group users (*P*<.001), but this association was non-significant when considering the number of favorited tweets per day since the account was created. Sleep group users had both significantly fewer followers per day as well as friends per day (*P*<.001 for both).

For tweet-level data, the day-of-week and time-of-day data analyses were performed on a subset of data for which user-submitted time zone data were available. For all compiled timeline tweets (n=418,773), 73.5% had user-submitted time zones for which time zone specific date and time tweet data could be calculated. There was a significant difference between sleep group and non-sleep group users in the proportion that did or did not have user-submitted time zone information; 76.8% of sleep group users disclosed their time zone while only 64.0% of others provided this data (*P*<.001).

**Figure 1:**
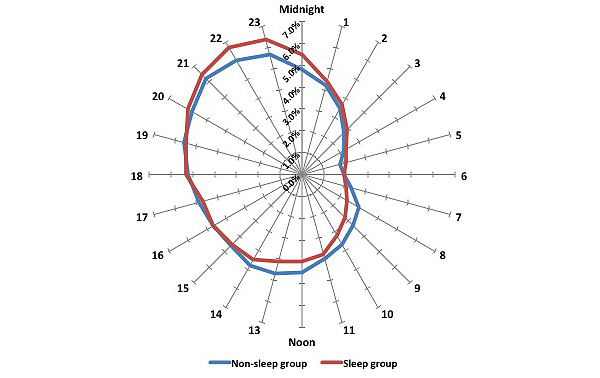
Proportion of statuses posted each hour by user group.

**Figure 2:**
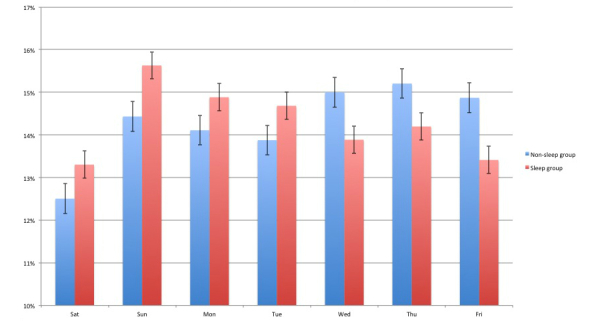
Proportion of statuses posted each day by user group. Y-axis begins at 10% to more clearly demonstrate differences between groups. All differences between groups were statistically significant (P<.001).

A larger proportion of tweets between 12 am-5:59 am were from sleep group users (*P*<.001), as well as between 6 pm-11:59 pm (*P*<.001 for both). Conversely, more tweets from between 6 am-11:59 am were from non-sleep group users (*P*<.001). An hourly proportion of statuses posted by both groups is presented in Figure 1. In addition, a larger proportion of tweets that were submitted on Saturday, Sunday, Monday, and Tuesday, were from sleep group users (*P*<.001), whereas tweets on Wednesday, Thursday, and Friday, were more often from non-sleep group users (*P*<.001) (Figure 2).

Statistical analysis of sentiment scores calculated by AMT revealed that sleep group users (ie, those that were identified as expressing symptoms consistent with sleep issues) had significantly more negative tweet sentiment than non-sleep group users (*P*<.001), and conversely, that non-sleep group users had significantly more positive sentiment in their tweets (*P*<.001). There was no difference between groups in the neutral sentiment category (*P*=.45). To control for inherent variations in sentiment that might exist between individuals who tweet relatively infrequently and those who tweet more, we also categorized individual users into either low- or high-volume tweeters, which was determined by dividing the group in two at the median number of tweets for the entire sample population. When categorized, significant differences were still found between groups, with non-sleep group users having significantly more positive sentiment in both low- and high-volume groups (*P*=.002 and *P*=.03, respectively) and sleep group users showing significantly more negative sentiment in both groups (*P*=.003 and *P*=.03, respectively). Similar results were found when groups were dichotomized by the number of friends and number of followers for each user.

Sentiment was calculated by averaging ratings from two separate workers. While this approach has been used widely in the literature for AMT sentiment analysis, we also sought to determine agreement between workers. Agreement percentage and Cohen’s kappa values were calculated for the top 10 most prolific workers (out of 144 workers in total), who rated a total of 13,170 tweets, which accounted for over 36 of all jobs. Taking into account the percentage of agreement based on random chance, AMT worker agreement was 65 compared to the expected agreement of 40, with a kappa value of .420 (*P*<.001), representing moderate agreement [44]. These values are expected to be lower than the actual level of agreement, owing to the fact that we were not able to investigate the actual agreement between all workers given the sheer volume of AMT workers and because the most prolific workers are not necessarily the most “accurate” workers.

## Discussion

### Principal Findings

This study demonstrates introductory evidence that individuals exhibiting signs of sleep issues on Twitter were significantly less active on the social media platform than other users, but they tweet more during traditional sleeping hours and exhibited more negative sentiment in the tweets they shared.

In spite of conjecture found in the popular media [45,46], having some type of sleep issue, as it was defined in this study, did not equate to increased activity on social media. This conclusion is supported by the findings that sleep group users in our study had lower median values for number of followers, number of friends (users followed), and average number of tweets per day, which all indicate sleep group users appear to be less active on this particular social network. It is worth noting, however, that we were not able to determine how active users are on Twitter in terms of “observing” the social network, that is, reading tweets and passively tracking other users, while not actually posting tweets of their own or officially following other users. This has the potential to skew the results, as a user may appear to be relatively inactive by our definitions but could potentially be more active in ways that are not recorded by Twitter. Interestingly, while sleep group users were less active than non-sleep group users based on our definitions, it was observed that they had accounts that were significantly older (based on the date of account creation to date of tweet identification), suggesting that users with sleep issues may be more likely than those with normal sleep patterns to start using a new social media tool, even if they are less active on it. Although the phenomenon has not been scientifically investigated, there exists the possibility that users may tend to become less active on a social media account the longer they hold the account, which could explain the older account life of sleep group users, while their activity tends to be lower than others. While there are additional studies in progress that aim to elucidate these associations more clearly, these are interesting findings that may potentially warrant a reversal of how social media usage and the demographics of its users are perceived.

The finding that sleep group users posted a significantly higher proportion of their tweets during midnight and 6 am suggests that our method of sleep group determination is effective, since this is a time when most people with normal sleeping patterns would be asleep (after adjusting for time zone). It is impossible to say, without detailed investigations of all tweets from selected users, whether or not users tweeting between midnight and 6 am may actually have some reason (perhaps the user works a night shift or has some other reason for being awake during this time), but the significance of the difference between the two groups suggests the method employed to distinguish between sleep group and non-sleep group users is effective.

This study also provides introductory evidence for the argument that people suffering from insomnia and similar sleep disorders may be at increased risk of psychosocial issues. Of note, previous studies in the field of psychology and data mining have been successful in quantitatively linking online social media use, negative sentiment, and depression using automated tools [47-50]. In particular, an earlier study also found a significant relationship between Twitter users who tweeted about insomnia and a negative sentiment of those users [51]. We found that, based on AMT sentiment analysis results, Twitter users identified as potentially experiencing sleep issues had significantly lower sentiment portrayed in their tweets, suggesting this group may be experiencing some type of psychosocial disorder. Interestingly, this finding is backed up by findings that sleep group users had fewer friends, fewer followers, and fewer interactions than other users, indicating some level of decreased online social interaction for this group. While the association is an interesting finding, it is preliminary and not conclusive, leaving much to be answered. However, these results provide an excellent starting point for a deeper investigation into the link between sleep issues, psychosocial issues, and social media usage, and warrant further investigation by more focused studies. A logical progression to further investigate these results would be to assess if the social dynamics of individuals are similar in their “real life” as in their online, social media life, or if the two areas differ significantly.

Given the nature of this study, it is worth briefly discussing the ethical, legal, and social implications of using Twitter data to conduct research on sleep disorders with potential links to psychosocial issues. Unlike other social network sites that restrict view permission of posts to approved friends, Twitter is a microblog with the sole purpose of allowing anyone to view content without prior approval. The privacy policy used by Twitter indicates that users consent to the collection, transfer, manipulation, storage, and disclosure of data that are public, while each user has the ability to change the privacy setting for their account. This study analyzed only tweets that were completely public (ie, no privacy settings were selected by the user). Thus, there was no expectation of privacy by the user. Public Twitter data are considered consistent with other existing public data sources, and as data are only passively analyzed in aggregate, this type of research is generally not considered to fall under the protections of human research. However, active data collection (eg, interaction directly with users) raises legitimate ethical, social, and legal concerns and should be conducted with appropriate caution and Institutional Review Board oversight.

### Limitations

While the results presented above suggest that further research into this field is warranted, they also must be considered and interpreted in light of several potential limitations. Most importantly, due to the cross-sectional nature of the study, it is not possible to determine causality in the significant relationships found between social media usage, sleep issues, and psychosocial findings. Additionally, there were several methods pertaining to the curation of non-sleep and sleep group tweets that may merit revision when pursuing future projects. During the curation process, the curator was presented with tweets to determine whether or not the tweet was related to any sort of sleep-related disorder or not. Candidate tweets were presented to the curator in order of tweet creation, with the most recently posted tweets appearing for curation before others. This method is not optimal as the tweets a curator is reading are dependent on the time of day that curation is taking place. That is, the list of tweets the curator was working on may have been different from those that would be seen if the curator was working at 9 am compared to 9 pm To avoid this potential bias moving forward, future analyses will involve curation from a random selection of tweets stored in the database (thus randomizing the time and day of each tweet). While the method used here may potentially bias the users selected, they should not affect the analyses performed on the tweet-level data, since those analyses take all a user’s tweets into consideration, and therefore the timing of the tweet identified as belonging to the sleep or non-sleep group is irrelevant.

Additional information would be useful for controlling for inherent differences in Twitter users. For instance, Twitter usage profiles may differ between users of different age, gender, or ethnicity. In future studies, it would be advantageous to collect this information in an attempt to control for these factors. This could be done either via algorithms designed to estimate these variables, by administering surveys to participants in a more interactive study, or by following a large subset of users before/after they suffer from self-described sleep issues. As well, user time zone information, which was used in conjunction with the time of tweet (recorded in UTC) to calculate the time of day a tweet was created, is a user-submitted variable and is therefore subject to potential data inaccuracies. While there are no studies that investigate the proportion of location fields that are accurately identified, we suspect it is highly likely that a user will appropriately choose their time zone (which is voluntary). However, there is the possibility that a user might indicate an incorrect time zone.

Non-sleep group users were defined by the absence of pre-defined keywords in a user’s previous 10 days of tweets. As described above, this length of time was chosen to be computationally and financially achievable, while still achieving the desired amount of data. In future studies, we intend to increase the length of time a user’s tweets must be free of these pre-defined keywords in order to be included in the non-sleep group. Depending on the quantity and quality of data available and the type of hypotheses involved, this may entail investigating months, years, or even a user’s entire timeline of tweets, in order for group status to be designated. This will also allow us to analyze and control for specific time periods in a user’s account history (eg, such as the first few months after account creation). Additionally, we may want to further characterize the sleep group population to determine if users who post “can’t sleep” are different than those who post about “melatonin” or medications, for example. This finer-grain characterization may result in multiple sleep groups that should be analyzed independently.

While the information gathered in this study is interesting, and caution was taken to ensure its validity, this type of data is observational and as such no cause-and-effect relationships can be assumed. We have found significant differences between a non-sleep group and individuals who we have been categorized as having some type of sleep issue; however, we cannot be sure that those individuals who fit our definitions do in fact have a sleep issue. This is an important factor that we hope to address in further studies, potentially by directly interacting with users to help confirm our categorization methods. However, this approach raises ethical, social, and legal concerns (as mentioned above) and would need to be carefully implemented.

We also recognize that there may be inherent differences between users that can be reflected in their number of friends, followers, status update frequency, location, and other metrics that we have not accounted for. In future work, we aim to control for this by either following a large number of users for a long period of time (before and after self-described sleep issues) or by using a matching technique to more reliably compare groups.

Despite its limitations, this study and others focusing on using social media applications for addressing issues of public health concern demonstrate that this type of research can add meaningful interpretations to traditional methods. It is worth noting that while we see great promise for these new methods, they are envisioned and designed to be used alongside more traditional, highly validated methods such as the BRFSS. Both traditional and emerging ways of collecting and analyzing public health information and relationships have their strengths and shortcomings. We hope that by marrying the two types of research we can gain a more complete and accurate view of the state of health in the population.

### Conclusions

This is one of the first research studies to actively investigate the relationship between social media use and sleep issues. It was found that people with apparent sleep issues were, on average, less active on Twitter and tended to be most active on the weekend and early weekdays, compared to users who did not have self-described sleep issues (based on our criteria). Additionally, we found that users with sleep issues have significantly more negative sentiment in the tweets they are posting compared to others, which may indicate a tendency for individuals identified as having a sleep issue via social media to be at a greater risk of psychosocial issues. While our findings are preliminary, they warrant further investigation and begin to provide evidence to contradict the popular belief that social media causes insomnia and other common sleep disorders. Furthermore, our current findings offer promise for expansion into the use of social media on the investigation of other health outcomes associated with sleep-related issues.

## Abbreviations

AMT: Amazon Mechanical Turk
BRFSS: Behavioral Risk Factor Surveillance System
UTC: Universal Time Code
